# Retraction: Heterologous expression, purification and characterization of nitrilase from Aspergillus niger K10

**DOI:** 10.1186/1472-6750-13-57

**Published:** 2013-07-20

**Authors:** Ondrej Kaplan, Karel Bezouska, Ondrej Plihal, Rudiger Ettrich, Natallia Kulik, Ondrej Vanek, Daniel Kavan, Oldrich Benada, Anna Malandra, Ondrej Sveda, Alicja B Vesela, Anna Rinagelova, Kristyna Slamova, Maria Cantarella, Jurgen Felsberg, Jarmila Duskova, Jan Dohnalek, Michael Kotik, Vladimir Kren, Ludmila Martinkova

**Affiliations:** 1 Institute of Microbiology, Academy of Sciences of the Czech Republic, Vídeňská 1083, CZ-142 20, Prague, Czech Republic; 2 Department of Biochemistry, Faculty of Science, Charles University in Prague, Hlavova 8, CZ-128 40, Prague, Czech Republic; 3 Centre of Biocatalysis and Biotransformation, Institute of Systems Biology and Ecology, Academy of Sciences of the Czech Republic, Zámek 136 373 33, Nové Hrady, Czech Republic; 4 Department of Chemistry, Chemical Engineering and Materials, University of L'Aquila, Via Campo di Pile - Zona industriale di Pile, I-67100, L'Aquila, Italy; 5 Institute of Macromolecular Chemistry, Academy of Sciences of the Czech Republic, Heyrovského náměstí 2, CZ-162 06, Prague, Czech Republic

## Retraction

The authors have retracted this article [1] due to the ethical misconduct of Karel Bezouska and misappropriation of data which fundamentally changes the main findings of the article. Specifically, the upper amino acid sequence in Figure 1 (by KB) in BMC Biotechnol **11**:2 was incorrect, therefore Nit-ANigWT and Nit-ANigRec (nitrilase expressed in *E. coli* harbouring a *nit* gene from *A. niger* K10) were not variants of the same enzyme as hypothesized in the original article. The different biochemical properties of Nit-ANigWT and Nit-ANigRec discussed in the paper were caused by a significant difference in the primary structures of these enzymes.

## References

[B1] Kaplan O Bezouska K Plihal O Ettrich R Kulik N Vanek O Kavan D Benada O Malandra A Sveda O Vesela AB Rinagelova A Slamova K Cantarella M Felsberg J Duskova J Dohnalek J Kotik M Kren V Martinkova L Heterologous expression, purification and characterization of nitrilase from Aspergillus niger K10 BMC Biotechnol 2011 11 2 10.1186/1472-6750-11-2 21210990 PMC3023689

